# Examination of the Dundee Ready Educational Environment Measure (DREEM) Model-Based Educational Quality in the Clinical Divisions of Semnan University: A Descriptive Study

**DOI:** 10.7759/cureus.54171

**Published:** 2024-02-14

**Authors:** Shaghayegh Pazoki, Maryam Hajiahmadi, Elham Saffarieh

**Affiliations:** 1 Medical School, Semnan University of Medical Sciences, Semnan, IRN; 2 Educational Development Office of Medical School, Semnan University of Medical Sciences, Semnan, IRN; 3 Abnormal Uterine Bleeding Research Center, Semnan University of Medical Science, Semnan, IRN

**Keywords:** clinical departments, internship, medical student, dreem model, educational environment

## Abstract

Introduction: It is widely recognized that there is a significant correlation between the quality of the learning environment and the level of satisfaction and achievement of students. To enhance the quality of education, it is vital to discern and rectify any inadequacies or deficiencies within the learning environment. Since the examination of strengths and weaknesses of the clinical learning environment provides valuable information to educational managers in improving the quality of education, the primary objective of this study was to examine the educational atmosphere prevalent in the principal clinical departments of the academic medical centers affiliated with Semnan University of Medical Sciences. The investigation was carried out based on the principles of the Dundee Ready Educational Environment Measure (DREEM) model.

Materials and methods: The present study was conducted with a descriptive-analytical approach which consisted of 232 trainees and interns affiliated with the departments of pediatrics, gynecology, internal medicine, and surgery. Sampling was done by census method. Then, a standard questionnaire was designed based on the DREEM model in Google Forum and its link was uploaded by representatives in class groups and channels. The data were collected within a period of three months and then were statistically analyzed using SPSS, version 24 (IBM Corp., Armonk, NY).

Results: Based on the results, the mean age of students was 23.92 years. The mean score of the educational environment of interns and stagers based on the questionnaire was 122.45 and 143.35, respectively. Regarding the mean score of the educational environment, a statistically significant difference was observed between pediatric and internal medicine (P-value<0.001), and surgical and internal medicine departments (P-value=0.03).

Conclusion: In general, the educational environment was evaluated as semi-optimal from students’ point of view; thus, it appears imperative for university officials to allocate greater attention toward enhancing the quality of the learning environment by devising more meticulous plans and engaging in consistent self-assessment of the educational landscape.

## Introduction

The investigation of the quality of the medical educational environment has attracted a lot of attention on a global scale [1]. The primary objective of universities is to prepare a skilled workforce based on society's requirements, promote knowledge and research, and provide an appropriate platform to facilitate the progress of developing nations. The educational environment plays a critical role in facilitating efficient learning [2]. Within the realm of medical education, the educational milieu is characterized by intricate intellectual challenges, making it one of the most intricate, exceptional, and specialized learning settings.

In fact, recognizing the importance of the educational environment is based on the information exchange and expansion of services [3]. According to various studies, 38% of the content is not transferred between students at the beginning of education, because educational programs are developed with the development of the educational environment [3]. Learning environments are made up of elements that become meaningful together. The quality and characteristics of these elements possess significant influence in shaping diverse behaviors. It is noted that favorable psychosocial attributes of the learning environment are strongly correlated with desirable learning outcomes, indicating that achieving optimal results is mainly dependent on providing an optimal learning environment [4,5].

The learning environment (atmosphere) can be different in every field of education; so it could be close to or away from the standard [5-7]. The educational environment is an expression of a curriculum and the spirit of an educational program. The learning environment is a behavioral determinant and the student’s perception of their surroundings regarding education [6,7].

Due to the lack of a written internship program, insufficient familiarity of professors with clinical and group teaching methods, as well as the unavoidable interference of healthcare services with education, the educational environment of clinical departments is relatively harsh and different from classroom education; therefore, upon arrival, students are not able to learn properly and adapt to the clinical environment [8]. Besides, a general comprehension of the learning environment is a crucial and necessary determinant of education. The present study employed the Dundee Ready Educational Environment Measure (DREEM) model as a tool to examine the educational environment quality. This model is used as a diagnostic tool for curriculum problems as well as the effectiveness of changes in education or identifying the difference between the real and ideal environment, which can provide valuable information to educational managers [9-12]. Its main characteristics include scientificity, practicality, sociality, suitability, and desirability [10]. Consequently, the current investigation was carried out to explore the quality of the educational environment prevalent in the main clinical departments of the academic medical centers affiliated with Semnan University of Medical Sciences, utilizing the DREEM model.

## Materials and methods

A descriptive-analytical research design was employed to examine the educational landscape within the clinical departments (internal medicine, surgery, pediatrics, and gynecology) of the academic medical centers affiliated with Semnan University of Medical Sciences, utilizing the DREEM model. The study sample, comprising 232 trainees and interns, was selected through the census method.

Data collection

This descriptive-analytical study was conducted on 232 students and interns in departments of internal medicine, surgery, pediatrics, and gynecology. We used the census method for sample size calculation. Then, a standard questionnaire was designed based on the DREEM model in Google Forum. Representatives in class groups and channels uploaded its link. The questionnaire adopted in this study comprised two distinct components: demographic characteristics (such as gender, age, period of experience, department, and year of admission) and DREEM subscales [12], encompassing 12, 11, 8, 12, and seven questions about students' perceptions of teaching, teachers, academic self-perception, atmosphere, and social self-perception, respectively. Respondents were required to rate their responses on a 5-point Likert scale, with options ranging from “I completely agree” (scored as 4) to “I completely disagree” (scored as 0). An experimental investigation was carried out with a sample of 25 students, utilizing Cronbach's alpha coefficient (r=0.77) to enhance the reliability of the questionnaire. To interpret the final score obtained from the questionnaire, four ranges, including non-optimal 0-50, semi-optimal 50-100, optimal 101-150, and highly optimal 151-200, were considered.

We comprehensively understood the educational setting by examining each individual's responses. Achieving a score of ≥3.5, ≤2, and 2-3 in each question signifies strength, weakness, and assessment of the academic environment, respectively.

Following a three-month data collection period, the data were analyzed using SPSS, version 24 (IBM Corp., Armonk, NY). We calculated the mean, standard deviation, and frequency distribution for data presentation. To compare numerical values, a t-test or one-way ANOVA with statistical bias was employed. For comparing numerical variables without statistical bias, Mann-Whitney U or Kruskal-Wallis tests were utilized. The Kolmogorov-Smirnov test was employed to verify normality, while Levene's test was used to confirm homogeneity (p=0.05). A P-value below 0.05 was set to indicate statistical significance.

Ethical considerations

This study was registered with the Ethics Committee of Semnan University of Medical Sciences under the assigned code No. 269/IEC/PGM/2021. We encouraged the participation of volunteers by protecting the privacy of their personal information.

## Results

This descriptive-analytical study was conducted on 232 stagers and interns in departments of internal medicine, surgery, pediatrics, and gynecology. As per the findings, the average age of the students was 23.92 years. The mean score of the educational environment of interns and stagers based on the questionnaire was 122.45 and 143.35, respectively. Out of 232 participants, 127 (54.7%) and 105 (45.3%) were female and male, respectively; 95 (40.9%) and 137 (59.1%) of them were stager and intern, respectively (Table 1). The frequency distribution of the subjects by department and gender is presented in Table 2.

**Table 1:** Demographic characteristics of the subjects

Variable		Total Number (N)	Percentage (%)
Gender	Female	127	54.7
	Male	105	45.3
Period of experience	Stager	95	40.9
	Intern	137	59.1
Department	Paediatric	58	25
	Surgery	58	25
	Internal medicine	77	33.2
	Gynaecology	39	16.8
Admission year	2013	2	0.9
	2014	55	23.7
	2015	92	39.7
	2016	41	17.7
	2017	29	12.5
	2018	13	5.6

**Table 2:** Frequency distribution of the subjects by department and gender N: Total number %: Perc

Period of experience	Gender	Department			
		Paediatric (N;%)	Surgery (N; %)	Internal medicine (N; %)	Gynaecology (N; %)
Stager	Female	6(2.6%)	9(3.9%)	28(12.1%)	10(4.3%)
	Male	10(4.3%)	14(6.0%)	13(5.6%)	5(2.2%)
Intern	Female	25(10.8%)	15(6.5%)	18(7.8%)	16(6.9%)
	Male	17(7.3%)	20(8.6%)	18(7.8%)	8(3.4%)

Through measuring Spearman's rank correlation coefficient (r=0.134, P-value=0.041), a notable correlation was detected between the overall score of educational environment quality and the age of the participants. As the age increased, a more positive attitude toward the educational atmosphere was observed. This weak correlation was observed only in academic self-perception (r=0.197, P-value=0.003); anyway, the study did not reveal a significant correlation between the quality of the educational environment and gender (r=-0.056, P-value=0.397), as demonstrated in Table 3.

**Table 3:** Dundee Ready Educational Environment Measure (DREEM) subscales statistics by department Mean ± SD: Mean and Standard Deviations, p<0.05 is considered significant

Sub scales					
	Paediatric Mean ± SD	Surgery Mean ± SD	Internal Medicine Mean ± SD	Gynaecology Mean ± SD	p-value
Perception of teaching	37 ± 4	34 ± 8	29 ± 9	33 ± 8	<0.001
Perception of teachers	32 ± 3	30 ± 5	27 ± 6	29 ± 5	<0.001
Academic self-perception	24 ± 3	22 ± 6	19 ± 7	21 ± 6	<0.001 (significant)
Perception of atmosphere	38 ± 5	35 ± 8	30 ± 9	34± 7	<0.001
Social self-perception	19 ± 4	18 ± 4	16 ± 5	17 ± 3	0.002
Total	150 ± 16	138 ± 29	121 ± 34	134 ± 26	<0.001

By performing follow-up and Bonferroni tests, a statistically significant difference was observed between pediatric and internal medicine (P-value<0.001), and surgical and internal medicine departments (P-value=0.03) regarding the mean score of educational atmospheres. Social self-perception had the lowest score among the subjects in different departments of the hospital. Also, perception of the atmosphere had the highest score among the students of the pediatrics, surgery, and gynecology departments.

Furthermore, a significant correlation was identified between the period of experience and the quality of the educational environment, with interns garnering higher scores across all five subscales. Thus, regarding the perception of teaching, the mean scores of pediatric and surgery interns were both 37±5, while the mean scores of pediatric and surgery stagers in terms of the same subscale were 36±4 and 28±8, respectively (Table 4).

**Table 4:** Dundee Ready Educational Environment Measure (DREEM) subscales statistics by period of experience and department Mean ± SD: Mean and Standard deviation

Subscales	Period of experience	Department				P-value
		Paediatric Mean ± SD	Surgery Mean ± SD	Internal Mean ± SD	Gynaecology Mean ± SD	
Perception of teaching	Stager	36±4	28±8	27±8	30±9	0.001 (significant)
	Intern	37±5	37±5	31±11	35±7	0.066
P-value		0.512	<0.001	0.033	0.212	
Perception of teachers	Stager	31±3	27±5	27±5	28±5	0.015 (significant)
	Intern	32±3	32±4	28±7	30±5	0.053
P-value		0.210	<0.001	0.199	0.076	
Academic self-perception	Stager	24±2	17±6	17±6	20±5	<0.001 (significant)
	Intern	25±4	25±5	21±8	22±6	0.097
P-value		0.62	<0.001	0.007	0.062	
Perception of atmosphere	Stager	37±4	30±8	27±8	33±6	<0.001 (significant)
	Intern	38±5	38±5	32±10	35±7	0.053
P-value		0.854	<0.001	0.028	0.383	
Social self-perception	Stager	18±4	16±4	15±4	17±3	0.050
	Intern	19±4	19±4	17±5	16±4	0.044 (significant)
P-value		0.631	0.005	0.068	0.999	
Total	Stager	147±13	118±29	113±28	127±26	<0.001 (significant)
	Intern	151±17	152±21	129±39	139±25	0.070
P-value		0.620	<0.001	0.035	0.172	

Based on the results, the educational environment in the pediatric department was optimal and highly optimal. About 10 (15.5%) and 48 (82.7%) of the subjects evaluated the educational environment in the surgery department as semi-optimal and highly optimal, respectively. A total of four (10.2%) and 35 (89.7%) of the subjects evaluated the educational environment in the gynecology department as semi-optimal and highly optimal, respectively. Nearly three (3.8%), 20 (27%), and 53 (70%) of the subjects evaluated the educational environment in the internal medicine department as non-optimal, semi-optimal, and highly optimal, respectively.

## Discussion

This descriptive-analytical study was conducted in departments of internal medicine, surgery, pediatrics, and gynecology. The mean score of the educational environment of interns and stagers based on the questionnaire was 122.45 and 143.35, respectively.

The findings suggest a notable correlation between the period of experience and the quality of the educational environment, with interns displaying higher scores across all five subscales. In a similar study by Mansorian, the mean score of the academic environment was 127.5 [11].

Numerous research have examined the effect of the quality of the learning environment on academic achievement, such as those conducted by Diggs [13] and Williams [14]. In a study by Hajiesmaello et al. [15], the average educational atmosphere score across key departments at Birjand University of Medical Sciences was 155.03. In the present study, interns had a higher score in all five subscales compared to stagers. These findings could be due to more involvement of students with clinical education, patients, and increased confidence of clinical professors in the students. Also, in both studies, the mean score of the clinical teaching in different departments had a significant statistical difference (p=.001), possibly due to the individual differences between professors and educational facilities in various departments.

The current investigation found that the perception of the atmosphere yielded the highest mean score. At the same time, social self-perception garnered the lowest mean score, which contradicts the findings of Daryazadeh et al.'s study [16]. Other research has had varying results. For instance, Mushtaq et al. found an inadequate educational environment, which contradicts our findings [17]. Therefore, the academic environment from the point of view of students in different regions according to the type of university, existing facilities, and clinical professors is evaluated as semi-optimal, and improving the educational environment through managers and officials is highly important.

The surgical department's educational environment was rated as semi-excellent and highly ideal by approximately 10 (15.5%) and 48 (82.7%) individuals, respectively. Four (10.2%) and 35 (89.7%) participants rated the teaching environment in the gynecology department as semi-excellent and highly ideal, respectively. Three individuals (3.8%), 20 (27%), and 53 (70%) rated the teaching environment in the internal medicine department as non-optimal, semi-optimal, or highly optimum, respectively.

The mean scores for several questionnaire items were low across all departments; for instance, there was no adequate structure to support stressed-out students or assistants, and there was a possibility of student abuse by professors and assistants. Maybe the internship's learning environment-aside from the pediatric department-needs to be examined. There is a need for educational interventions because other departments have not done well in student-centered teaching. We can list the relationship between professors and students, students' prior knowledge and understanding of the fundamentals of patient care in other hospital departments, students' interests, and the facilities of different hospital departments as additional explanations for the disparities in attitudes toward various departments.

One of this study's most important strengths is the use of the census sampling method, which guarantees a suitable sample size. Additionally, the brief data-collecting period was designed to minimize missing data and lower the likelihood of errors. Our cross-sectional study has some limitations, including a small sample size and only recruited students from four departments.

## Conclusions

In conclusion, students rated the educational atmosphere as semi-optimal. The findings suggest that certain areas, such as internal medicine, deserve additional attention from university administrators and management. As a result, university authorities must prioritize improving the quality of the learning environment through careful planning and ongoing self-evaluation of the educational landscape.

## References

[1] Al-Naggar RA, Abdulghani M, Osman MT (2014). The Malaysia DREEM: perceptions of medical students about the learning environment in a medical school in Malaysia. Adv Med Educ Pract.

[2] Mehrdad M, Mahfoozi L, Samipoor M, Samipoor F (2016). Medical students’ perception of educational environment of teaching hospitals of Guilan University of Medical Sciences in 2015. J Med Edu.

[3] Sameri M, Alilu L (2021). Evaluation of clinical educational environment based on PHEEM model from residents’ viewpoints in Urmia University of Medical Sciences in 2020. Nursing Midwifery J.

[4] Mihalko BJ (2010). The influence of transfer system factors and training elapsed time on transfer in a healthcare organization. Wayne State Univ Diss.

[5] Genn JM (2001). AMEE Medical Education Guide No. 23 (Part 1): Curriculum, environment, climate, quality and change in medical education-a unifying perspective. Med Teach.

[6] Hakimi S (2019). A century (1919-2019) of academic midwifery in Iran: from traditional midwives to PhD graduates. Eur J Midwifery.

[7] Pourshirazi M, Heidarzadeh M, Taheri M, Esmaily H, Babaey F, Talkhi N, Gholizadeh L (2022). Cesarean delivery in Iran: a population-based analysis using the Robson classification system. BMC Pregnancy Childbirth.

[8] Bahri N, Tabatabaeichehr M, Roudsari RL (2018). Comparative assessment of Iranian midwifery education curriculum against the International Confederation of Midwives (ICM) global standards for midwifery education. FMEJ.

[9] Hajifoghaha M, Nahidi F, Alizadeh S, Golezar S, Dabiri F, Mokhlesi SS, Babaei A (2020). Midwives’ educational needs in Iran: a narrative review. Iran J Nurs Midwifery Res.

[10] Poursamad A, Zahedi RM, Mohammad T, Nouraleh S, Shekarpour A (2021). Investigating the educational environment of teaching hospitals from the point of view of clinical students and its relationship with managers' performance. Armaghane Danesh.

[11] Mansorian A (2013). Views of learners on learning environment based on the model DREEM in Golestan University. JMED.

[12] Farahmand S, Bagheri-Hariri S, Moghanloo S, Basir Ghafouri H, Saeedi M, Afzalimoghadam M, Gao Y (2014). Evaluating the quality of the educational environment for medical interns in an emergency department using the DREEM inventory. Acta Med Iran.

[13] Diggs BK (2011). Perception of transfer climate factors in the macro and micro organizational work environment.

[14] Williams DJ (2008). An analysis of the factors affecting training transfer within the work environment. Theses Diss.

[15] Hajiesmaello M, Hajian S, Riazi H, Majd HA, Yavarian R (2022). Challenges facing clinical midwifery education in Iran. BMC Med Educ.

[16] Daryazadeh Y, Azadchehar S, Akbari R (2020). Evaluation of the clinical environment from the point of view of medical students of Kashan University of Medical Sciences based on the DREEM model in 2017. Horizons Med Educ Develop.

[17] Mushtaq R, Ansar A, Bibi A (2017). Quality of educational environment at Wah Medical College: assessment by using Dundee ready educational environment measure. J Ayub Med Coll Abbottabad.

